# EGb 761^®^ Does Not Affect Blood Coagulation and Bleeding Time in Patients with Probable Alzheimer’s Dementia—Secondary Analysis of a Randomized, Double-Blind Placebo-Controlled Trial

**DOI:** 10.3390/healthcare9121678

**Published:** 2021-12-03

**Authors:** Charlotte Kloft, Robert Hoerr

**Affiliations:** 1 Institute of Pharmacy, Freie Universität Berlin, 12169 Berlin, Germany; charlotte.kloft@fu-berlin.de; 2 Research & Development, Dr. Willmar Schwabe GmbH & Co. KG, 76227 Karlsruhe, Germany

**Keywords:** Ginkgo, EGb 761^®^, blood coagulation, bleeding time, acetylsalicylic acid, warfarin, interaction, safety, randomized controlled trial, Alzheimer’s disease

## Abstract

Following reports of bleeding upon Ginkgo intake, we assessed whether Ginkgo extract EGb 761^®^ affects coagulation or platelet function or increases the risk of bleeding. In a double-blind, placebo-controlled trial, prothrombin time, activated partial thromboplastin time, international normalized ratio and bleeding time were measured in patients with Alzheimer’s dementia at baseline, weeks 6 and 26. A total of 513 patients were randomized to 120 mg (*n* = 169) or 240 mg EGb 761^®^ (*n* = 170) or placebo (*n* = 174). No relevant changes were found for coagulation parameters and bleeding time. Numbers of bleeding-related adverse events were similar in all groups. Concomitant intake of acetylsalicylic acid was documented for 68 patients in the placebo group and 105 in the EGb 761^®^ groups. Within these groups, the means at baseline and week 26 differed by less than 1 unit for prothrombin time and bleeding time and less than 0.1 unit for international normalized ratio. Data on warfarin treatment in nine patients each taking placebo or EGb 761^®^ did not indicate enhancement of warfarin effects by EGb 761^®^. No evidence was found that EGb 761^®^ affects hemostasis or increases the bleeding risk. No pharmacodynamic interactions with warfarin or acetylsalicylic acid were found.

## 1. Introduction

Analyses of aggregate spontaneous reports from the US and Australia conclude that the intake of Ginkgo preparations may lead to an increased risk of bleeding due to interactions with warfarin [1,2]. Individual published case reports suspect bleeding due to concomitant use of Ginkgo extract with acetylsalicylic acid (ASA).

The retrospective analysis of a large database (Veterans Administration Informatics and Computing Infrastructure, VINCI) in the US addressed the risk of bleeding in patients on warfarin who concomitantly took “Ginkgo” as a food supplement. The results were interpreted as suggesting that taking Ginkgo preparations concomitantly with warfarin increases the patient’s risk of a bleeding-related adverse event [2]. 

A descriptive analysis of spontaneously reported adverse drug reactions (ADR) to Ginkgo biloba from 2000 to 2015 was performed in the electronic database of the Therapeutic Goods Administration in Australia [1]. The authors ascribed three adverse drug reactions to pharmacokinetic interactions with warfarin. Their assumption is based on research on hepatocytes, showing that Ginkgo induced the cytochrome P450 3A4 enzyme which may affect the R enantiomer of warfarin [3]. 

To further elucidate the potential of EGb 761^®^ to affect blood coagulation or cause bleeding events or pharmacodynamic interactions with warfarin and/or ASA, we analyzed still-unpublished safety data from an already reported double-blind, placebo-controlled trial [4]. Data were provided by the sponsor of the trial (Dr. Willmar Schwabe GmbH & Co. KG, Karlsruhe, Germany). The study was carried out in elderly patients in the US with probable Alzheimer’s disease. Prothrombin time (PT), activated partial thromboplastin time (APTT) and international normalized ratio (INR) were performed in all patients. Bleeding time (BT) tests were performed at clinical sites with qualified staff. Furthermore, the number of bleeding events as suspected adverse drug reactions was analyzed compared to placebo. 

## 2. Materials and Methods

Methodological details, a description of the patient sample and the main results of the trial have been published elsewhere [4]. The study was conducted in accordance with the Declaration of Helsinki and the Good Clinical Practice guidelines of the International Council for Harmonization. The trial was approved by the Institutional Review Boards of study principal investigator’s site and all participating clinical sites. Patients were provided with oral and written information about the purpose, objective, procedures and risks of the trial, and were enrolled only after written informed consent was obtained from the patient, and if appropriate, a legally authorized representative.

### 2.1. Patients

Outpatients were recruited by 44 clinical sites including university hospitals, private practices and research institutes specialized in neurology or psychiatry across the United States. The participants were at least 60 years of age and were enrolled if they had probable dementia of the Alzheimer’s type in accordance with the diagnostic criteria of the National Institute of Neurological and Communicative Disorders and Stroke and the Alzheimer’s Disease and Related Disorders Association, NINCDS/ADRDA [5], with mild-to-moderate severity (scoring 10 to 24 on the Mini-Mental State Examination, MMSE [6].

### 2.2. Study Medication

The study drug EGb 761^®^ is a dry extract from Ginkgo biloba leaves (drug extract ratio 35–67:1, extraction solvent: acetone 60% (*w/w*)) in compliance with the quality standards of the European Pharmacopoeia. The extract is adjusted to 22–27% Ginkgo flavonoids calculated as Ginkgo flavone glycosides and 5–7% terpene lactones consisting of 2.8–3.4% ginkgolides A, B, C and 2.6–3.2% bilobalide and contains less than 5 ppm ginkgolic acids. Film-coated tablets as well as placebo tablets were manufactured and supplied by Dr. Willmar Schwabe GmbH & Co. KG, Karlsruhe, Germany, the sponsor of the trial. Drug and placebo tablets were indistinguishable by size, shape and colour.

Concomitant treatment with antidementia medications or other drugs acting on the central nervous system was not permitted during the study period. Patients who needed anticholinergic and psychoactive drugs, such as antidepressants, antipsychotics, hypnotics, and tranquilizers, except for occasional doses of short-acting benzodiazepines for restlessness or sleep disturbances, were excluded from the study. 

The concomitant intake of nonsteroidal anti-inflammatory drugs was permitted at doses that were stable for at least 6 months. Other comedication potentially affecting the coagulation, i.e., warfarin or coagulation disorders, were not excluded. In warfarin-treated patients, the INR was watched closely and kept within the prespecified range (usually between 2.0 and 3.5, depending on the primary physician’s orders), by adapting the dosage regimen of warfarin if necessary. 

### 2.3. Study Design

After a medication-free four-week period, patients underwent the baseline examination and were randomized to one of three treatment groups taking 120 mg or 240 mg EGb 761^®^ (60 mg or 120 mg twice daily) or placebo for 26 weeks. Study drug and placebo were packed and labelled in an indistinguishable manner according to the randomization list, and the investigators as well as the whole study team remained blinded with respect to the treatment assignment until final lock of the database. Follow-up examinations were scheduled at 6, 12, 18 and 26 weeks after baseline. Standard outcome variables were employed to assess efficacy in dementia. Safety was evaluated by means of laboratory tests and by assessment of adverse events (AEs) that occurred after intake of the first dose of study medication. 

### 2.4. Blood Coagulation and Bleeding Time Tests

PT [7], APTT [8] and INR [9] were measured at baseline and after 6 and 26 weeks of treatment. Venous blood (4.5 mL) was collected in silicon-coated tubes containing 0.5 mL of buffered 3.2% sodium citrate, mixed gently by inverting the tube ten times and sent by courier to the central laboratory (SmithKline Beecham Clinical Laboratories, Van Nuys, California, USA) at ambient temperature. Coagulation tests were performed by validated automated procedures in accordance with laboratory standards. 

Measurement of BT was performed at all 44 sites which were able to comply with Clinical Laboratory Improvement Amendments (CLIA) as the US federal regulatory standards for clinical laboratories. 

BT to test platelet function was measured in an area on the lateral aspect of the volar surface of the forearm, approximately 5 cm distal to the antecubital fossa, employing the established Simplate^®^ procedure [10]. After local disinfection, a sphygmomanometer cuff was placed on the upper arm and inflated to 40 mm Hg. This pressure was maintained throughout the procedure. A standardized incision 5 mm long and 1 mm deep was then made with the Simplate^®^ device. The flowing blood was wicked with filter paper at regular intervals of 30 s. The filter paper was brought close to the incision, but care was taken not to touch the wound and the forming platelet clot. BT was determined as the time when blood no longer stained the filter paper, rounded up to the nearest 30 s time point.

### 2.5. Statistical Analyses

Descriptive statistics were calculated for the blood coagulation parameters and BT at baseline, after 6 and 26 weeks of treatment. These sample characteristics together with the patients’ individual values were assessed for relevant changes during treatment at the level of treatment groups as well as individual patients. The analyses are based on the intention to treat (ITT) analysis set. If blood coagulation parameters or BT of patients who dropped out prematurely were available at the end of the individual treatment period, these values were used for the analyses. Missing values were not replaced. This is a conservative approach for safety analyses since it can be expected that treatment effects are not underestimated.

Separate analyses were performed for three groups of patients:

(1) Those taking neither ASA nor warfarin as concomitant medication;
(2) Those taking ASA concomitantly;
(3) Those taking warfarin concomitantly.

Within the first two groups, coagulation parameters and BT were compared between active treatment groups and placebo and between 120 mg EGb 761^®^ and 240 mg EGb 761^®^ for each timepoint separately using *t*-tests. Since the third group was very small, the outcomes were compared between patients treated with EGb 761^®^ (120 or 240 mg) and placebo using *t*-tests. Two-sided *p*-values < 0.05 were considered statistically significant; *p*-values ≥ 0.20 were regarded as a hint that there were no relevant differences between the compared treatment groups. The threshold of 0.2 is used since this indicates that the type-one error rate for concluding an increased risk is at least 20%. 

Three patients (two patients from the placebo group and one from the EGb 761^®^ high-dose group) took both ASA and warfarin; thus, the group was too small to be evaluated separately. No bleeding event was observed in the individual patient taking EGb 761^®^. This patient’s values for PT, APTT and INR showed some fluctuation around the upper limits of the reference ranges. As a conservative approach, these three patients were added to both subgroups per concomitant medication. 

Furthermore, the analysis also included the number of bleeding events suspected to be adverse drug reactions in patients taking EGb 761^®^ versus placebo as well as the number of patients with measures above the upper limit of the reference range assessed as being clinically relevant by the investigator. The numbers of patients with bleeding events were compared between the treatment groups using the two-sided Mantel Heanszel chi-squared test.

## 3. Results

In total, 513 patients were randomized to take either 120 mg (*n* = 169) or 240 mg EGb 761^®^ (*n* = 170) or placebo (*n* = 174). At baseline, blood coagulation tests (PT, APTT, INR) of 499 patients could be analysed. BT measurements at baseline were available from 278 patients. Table 1 presents the demographic data of patients by concomitant use of anticoagulants and treatment group. Of the patients randomized, 20% (placebo: 22.4%, EGB 761^®^ 120 mg: 20.1% and EGb 761^®^ 240 mg 17.6%) dropped out prematurely and 80% completed the trial as scheduled. The following reasons were documented in the treatment groups (placebo/EGb 761^®^ 120 mg/EGb 761^®^ 240 mg): withdrawal of informed consent (*n* = 13/9/10), AEs (*n* = 7/8/10), SAEs (*n* = 3/3/5), lack of efficacy (*n* = 3/8/2) and other reasons (*n* = 13/6/3).

Intermittent use of nonsteroidal anti-inflammatory drugs (NSAIDs), selective serotonin reuptake inhibitors (SSRIs) or serotonin and noradrenaline reuptake inhibitors (SNRIs), which might affect platelet aggregation or bleeding time, was similar in all treatment groups: 60, 59 and 66 cases of NSAID use and 2, 2 and 1 cases of SSRI/SNRI use in the placebo, 120 mg and 240 mg EGb 761^®^ groups, respectively, were documented. Three patients in the placebo group and two patients in the 120 mg group were treated with heparin for short periods of time. One patient in the 120 mg group had thrombocytopenia at baseline.

### 3.1. Group Comparison without Concomitant Use of Anticoagulants

**Figure 1:**
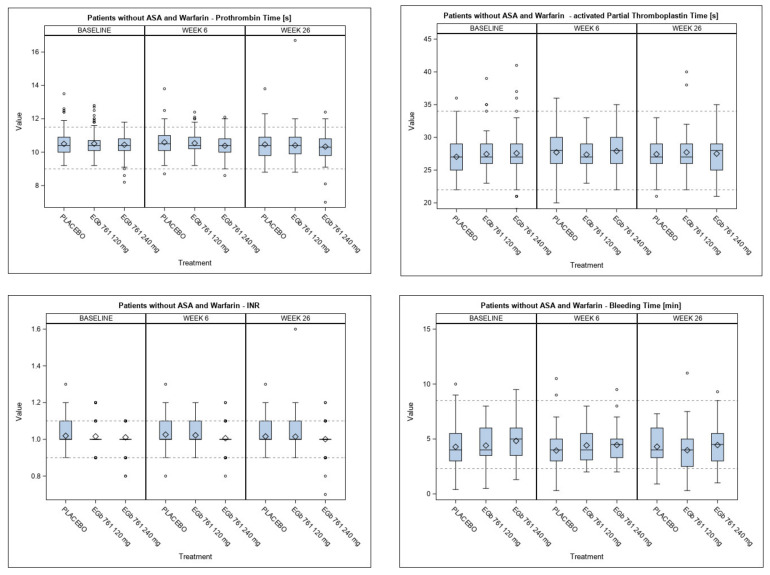
Blood coagulation (prothrombin time [reference range of 9–11.5 s], activated partial thromboplastin time [reference range of 22–34 s], international normalized ratio (INR) [reference range of 0.9–1.1]) and bleeding time [reference range of 2.3–8.5 min]) in subjects taking neither acetylsalicylic acid (ASA) nor warfarin as concomitant medication at baseline, 6 weeks and 26 weeks (endpoint). Box-and-whiskers plots showing the mean (diamond), median (line within the box), interquartile range [IQR] (box), range within 1.5xIQR (whiskers), values without 1.5xIQR (circles). All *p*-values for pairwise comparisons between the active treatment groups and placebo at week 26 are not significant.

Compared to placebo, no prolongation of PT, APTT or BT and no increase in INR was observed after 6 and 26 weeks of treatment with EGb 761^®^ (both doses) (Table 2). At both time points, the interindividual variabilities in the active treatment groups were similar to the variability in the placebo group (Figure 1). At baseline, there were no statistically significant differences between the active treatment groups and placebo (BT, *p* = 0.145/APTT, *p* = 0.195 for the comparison between 240 mg EGb 761^®^ and placebo; *p* > 0.200 for all other comparisons; see Table 2). From baseline to week 26, the mean values did not change relevantly within the treatment groups (Figure 1, Table 2). Accordingly, mean coagulation parameters and bleeding times were neither statistically significantly nor clinically relevantly higher in the active treatment groups than in the placebo group at week 26 (*p* > 0.200 for all pairwise comparisons between active treatment groups and placebo group; Table 2). There was no dose-dependent effect of EGb 761^®^ on any parameter of blood coagulation or BT (Figure 1 and Table 2).

### 3.2. Group Comparison per Concomitant Intake of ASA or Warfarin

Overall, 68 of the patients in the placebo group and 105 in the EGb 761^®^ groups took concomitant ASA, mostly for stroke prevention or as an analgesic. The median dose of ASA was 325 mg per day (range 81–700 mg) in the placebo group at baseline (*n* = 67), 200 mg (range 81–1000 mg) in the group treated with 120 mg EGb 761^®^ (*n* = 53), and 325 mg (range 81–650 mg) in the group taking 240 mg EGb 761^®^ (*n* = 48). Overall, more than 90% in each group were taking less than 500 mg ASA per day. 

**Figure 2:**
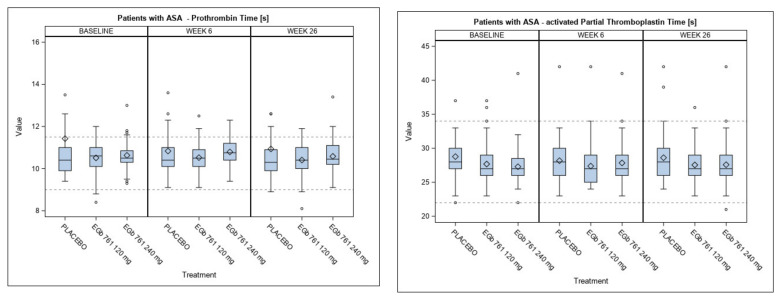

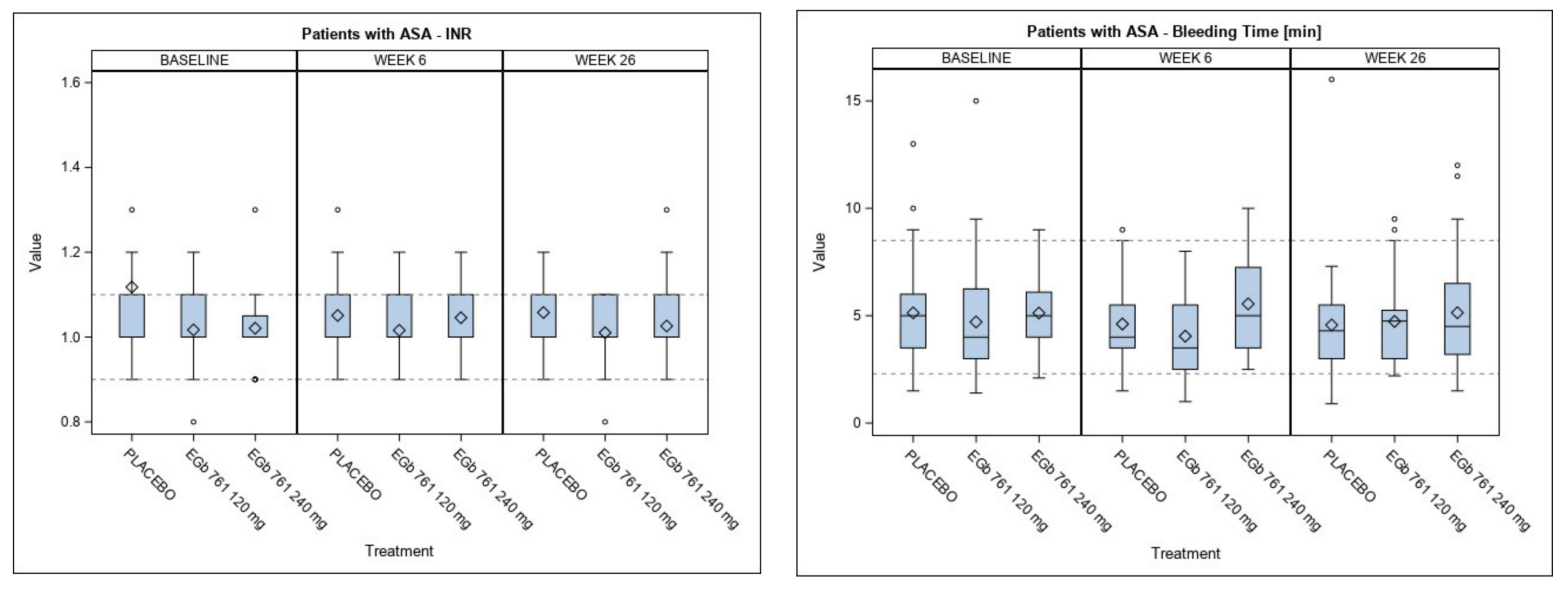
Blood coagulation (prothrombin time [reference range of 9–11.5 s], activated partial thromboplastin time [reference range of 22–34 s], international normalized ratio (INR) [reference range of 0.9–1.1]) and bleeding time [reference range of 2.3–8.5 min]) in subjects taking acetylsalicylic acid (ASA) as a concomitant medication at baseline, 6 weeks and 26 weeks (endpoint). (In the placebo group, two subjects who took acetylsalicylic acid and warfarin had values of PT, APPT and INR higher than 16 s, 50 s and 1.6, which are not displayed. These values were included in the calculation of the characteristics of the empirical distributions shown in the box plots.). Box-and-whiskers plots showing the mean (diamond), median (line within the box), interquartile range [IQR] (box), range within 1.5xIQR (whiskers), values without 1.5xIQR (circles). All *p*-values for pairwise comparisons between the active treatment groups and placebo at week 26 are not significant.

At baseline, the mean/median value of 98 patients with available values showed a prolonged bleeding time as expected from the pharmacological action of ASA (Table 3, in comparison to Table 2). After 6 and 26 weeks of treatment, the interindividual variabilities in the active treatment groups were equal to the variability in the placebo group. From baseline to week 26, the mean bleeding times in patients taking ASA in the placebo group and the EGb 761^®^ groups showed no relevant differences. Obviously, EGb 761^®^ did not enhance the antiplatelet effects of ASA (Figure 2). At baseline, PT, INR and bleeding times were similar in the active treatment groups and the placebo group (*p* = 0.162 for the comparison of INR between 120 mg EGb 761^®^ and placebo, *p* ≥ 0.200 for all other comparisons of these parameters). The means of APTT were slightly lower (1.1 and 1.5 s) in the active treatment groups than in the placebo group (*p* = 0.196 and 0.094). 

Pairwise comparisons showed that PT and INR at week 6 and week 26 were not statistically significantly higher in the active treatment groups compared to the placebo group (*p* = 0.189 for the comparison of PT between 120 mg EGb 761^®^ and placebo with a higher mean in the placebo group, *p* ≥ 0.200 for the other comparisons at week 26). At the end of the active treatment period, the mean APTT of patients treated with 120 mg and 240 mg EGb 761^®^ were 1.06 and 1.03 s shorter, respectively, than in the placebo group (*p* = 0.083 and *p* = 0.127). These differences were not clinically relevant, and they were already present at baseline. In the high-dose group, the bleeding time, which was slightly higher than in the low-dose group already at baseline, increased somewhat until week 6, but returned to baseline level until week 26. Minor differences between the two doses of EGb 761^®^ were observed after 6 weeks of treatment for PT and INR which were statistically significant but not clinically relevant. There were no consistent dose- or time-dependent changes, and after 26 weeks of treatment there was no dose-dependent effect of EGb 761^®^ on any parameter of blood coagulation or BT (Figure 2 and Table 3). 

A total of nine patients taking placebo and nine patients taking EGb 761^®^ received warfarin as a concomitant drug. They showed higher INR and prolonged PT and APTT (Table 4), than those patients without anticoagulant medication at all time points (Table 2). As shown in Table 4, mean values of PT, APTT and INR at week 26 did not differ relevantly from mean baseline values within each treatment group. At baseline as well as in week 26, there is no statistically significant difference between patients treated with EGb 761^®^ (both doses) and placebo (Table 4). The mean values of PT, APTT and INR were lower in the active treatment groups compared to the placebo group. However, the patient group was relatively small. Warfarin dosages remained unchanged during the study period except for two patients on placebo who needed a dose decrease and for one patient taking 120 mg EGb 761^®^ for whom a dose increase was reported. These observations do not indicate an enhancing effect of EGb 761^®^ on warfarin. 

### 3.3. Bleeding as an Adverse Event

In total, 37 bleeding events affecting 30 patients were documented in the treatment groups (Table 5). Fourteen events affected 10 of 174 patients (5.7%) in the placebo group, 10 events occurred in 8 out of 169 individuals (4.7%) in the 120 mg EGb 761^®^ group, and 13 events were documented in 12 out of 170 persons (7.1%) in the 240 mg EGb 761^®^ group. There is no hint of an increased risk of bleeding events for patients treated with EGb 761^®^ (*p* = 0.61, two-sided Mantel-Haenszel chi-squared test).

Of these events, the majority occurred in patients who took at least one concomitant drug with known anticoagulant side effects. Only eight patients in the EGb 761^®^ groups (two in the 120 mg daily dose group, six in 240 mg) were affected by bleeding events, for whom no concomitant anticoagulant drug exposure was documented. However, in six of them, evident other causes such as invasive cancer or fall- or accident-related injuries were present, and they were not assessed as causally related to the study medication. 

The investigator assessed seven bleeding events in five patients (*n* = 4 in the EGb 761^®^ groups, *n* = 1 in the placebo group) as potentially related to the study medication. Of these, only the individual patient in the placebo group did not take any concomitant medication with a known anticoagulant effect. 

A few test results that were above the reference range were assessed by the investigators as being clinically relevant. One patient taking 120 mg EGb 761^®^ had a low platelet count. In two cases (one on placebo at week 26, one on 240 mg EGb 761^®^ at week 6) these events were due to lab errors. In one patient who had discontinued 120 mg of EGb 761^®^ long before, this was due to a serious adverse event (hepatitis) not related to the study drug; and two cases (one patient on placebo at different time points, one on 120 mg EGb 761^®^ at baseline) the event was related to warfarin treatment.

Eleven bleeding events in 7 of 68 patients taking ASA and placebo were recorded, two bleedings in 2 of 57 patients taking ASA and 120 mg EGb 761^®^ and four bleeding events in 3 of 48 patients taking ASA and 240 mg EGb 761^®^. Bleeding-related adverse events were not documented in any of the patients in the warfarin group. Slight changes in coagulation parameters or bleeding time were considered to be not clinically relevant by the investigators. 

## 4. Discussion

The defined Ginkgo biloba leaf extract EGb 761^®^ is approved in many countries for the treatment of dementia. In randomized, placebo-controlled trials and meta-analyses, EGb 761^®^ was found to improve cognition, activities of daily living, overall condition and neuropsychiatric symptoms in patients with dementia [11,12,13]. 

Laboratory tests of blood-clotting parameters (PT, APTT, INR) with well-established methods revealed no influence of EGb 761^®^ on the extrinsic and intrinsic coagulation system in 499 elderly patients with probable dementia of the Alzheimer’s type. PT determines the clotting tendency of blood by measuring the time until coagulation after addition of tissue factor and evaluates the extrinsic pathway. The INR was devised to standardize the results of test systems for PT and is used to monitor the anticoagulant effect of warfarin. The APTT measures the time for plasma to coagulate after activation and evaluates the intrinsic pathway. 

Our data from 278 patients also showed that bleeding time remained unaffected by EGb 761^®^. Overall, the group comparisons of laboratory parameters and adverse events revealed no antiplatelet or other inhibitory activity of EGb 761^®^ on the haemostatic system as potential for pharmacodynamic interactions with anticoagulant drugs. In healthy persons, the BT measured with the Simplate^®^ method is up to 4 min. Under ASA therapy at steady state, the bleeding time increases by about 30% [14], which is the same range as determined in this study. Lifestyle factors such as smoking or drinking coffee may have an impact on platelet function and thereby on the coagulation process. During the study there was no control of such lifestyle factors which may be a limitation. However, we consider the magnitude of these effects small and probably equally affecting all treatment groups. Consequently, the risk of a bias is negligible.

These findings corroborate and add to the evidence derived from other studies and meta-analyses addressing the safety of EGb 761^®^ when taken alone or in combination with ASA or warfarin. They are consistent with results of two studies with 50 and 32 volunteers, respectively, in which no clinically significant effects of EGb 761^®^ on parameters of blood coagulation, platelet function or BT were found [15,16]. Another study also found no consistent change in APTT in a sample of 216 dementia patients randomized to receive either 240 mg EGb 761^®^ or placebo [17]. Likewise, no clinically relevant changes in APTT or INR were detected in a meta-analysis of clinical trials about EGb 761^®^ [18]. In a safety meta-analysis of 44 randomized placebo-controlled trials of EGb 761^®^ involving 3323 patients, the number of bleeding events documented as adverse events was not statistically different from that in the placebo group [19]. In two long-term, placebo-controlled trials with EGb 761, no increased bleeding risk was observed for treatments lasting five to six years [20,21]. Cumulative bleeding incidence was around 10% in these samples of elderly subjects with no difference between placebo and EGb 761^®^. Overall, the clinical database does not provide evidence that EGb 761^®^ causes pharmacodynamic effects on the coagulation system. However, drugs which are not coagulation-inhibiting themselves may affect the metabolism or elimination of anticoagulant substances and thereby lead to bleedings. This was discussed for EGb 761^®^ as well. However, no evidence for clinically relevant pharmacokinetic interactions was found so far [22]. 

A systematic review of the safety of concurrent use of food, herbal or dietary supplements and warfarin found 149 articles reporting 78 herbs (including Ginkgo biloba) which interact with warfarin [23]. From the analysis of time-at-risk assumptions in a large database, a signal for an interaction between warfarin and Ginkgo preparations was found. The authors developed a software to use within the natural language of the physicians’ notes and to detect statements that were consistent with current use of or a recommendation for use of Ginkgo. Unfortunately, the specificity of the method was only 87% (i.e., 13% of those identified as Ginkgo users were false positives), and dosage, beginning and end of Ginkgo product use was not further specified. The authors state that, ignoring time at risk, a bleeding event was observed in 3.4% of patients of the warfarin + Ginkgo group and in 5.4% of patients of the warfarin-only group [2]. This does not point to an increased risk of bleeding with Ginkgo. Another analysis of electronic health records of more than 42,000 warfarin-treated patients in the US did not find evidence that the use of Ginkgo preparations as dietary supplements led to bleeding events [24]. 

In our study, only very few patients took warfarin concomitantly, which does not allow us to draw definitive conclusions. These patients showed a markedly prolonged PT and APTT as well as an increased INR. In the placebo group, the decreased maximum value reflected the dose reduction in two patients. No attenuation of warfarin doses was required in EGb 761^®^-treated patients, which indicates that the Ginkgo extract did not enhance the effects of warfarin. In line with these observations, another study reported no effect of EGb 761^®^ on the clotting status or pharmacokinetics or pharmacodynamics of warfarin [25]. The information that three individual cases of bleeding after use of Ginkgo were reported spontaneously between 2000 and 2015 [1] cannot be evaluated, since there is no control group. Sometimes an inhibitory effect on platelet-activating factor (PAF) is assumed as the basis for this presumed mechanism of EGb 761^®^. PAF is characterized—among other effects—by an induction of rabbit platelet aggregation and is therefore detected in an assay with rabbit platelets. Of note, at least a 200-fold higher PAF concentration is required to induce aggregation of human platelets compared with rabbit platelets. This alone makes the involvement of PAF in human physiological platelet aggregation highly unlikely; nor has it been scientifically demonstrated to date.

It is noteworthy that the bleeding time seen in the 168 patients taking ASA was not affected by concomitant treatment with EGb 761^®^, i.e., the Ginkgo extract did not increase the platelet-inhibiting effect of ASA (see Table 3 and Figure 2). This is in keeping with the results of a randomized, double-blind, placebo-controlled 4-week study in older adults at risk of cardiovascular disease [26]. In this study, participants with peripheral artery disease or cardiovascular disease risk took either 300 mg of EGb 761^®^ or placebo in combination with 325 mg acetylsalicylic acid (ASA) daily. The platelet function analysis in 26 participants who took placebo and in 29 participants who took EGb 761^®^ showed no detectable effect on time for clotting and platelet aggregation. A further study with 100 male patients taking 500 mg ASA daily for 7 days also found no effect of EGb 761^®^ on coagulation and bleeding time [27]. So far, there is no clinical evidence that EGb 761^®^ affects platelet aggregation inhibitors binding to the P2Y12 receptor, i.e., clopidogrel.

ASA as well as warfarin are associated with the risk of additional major or minor bleeding events. The vitamin K inhibitor warfarin effectively prevents stroke, but it has a narrow therapeutic range, is subject to drug and food interactions and requires regular monitoring of the INR, resulting in the frequent need of dose adjustment. Annual rates for any bleeding in patients taking warfarin is about 5%. In inpatient and outpatient settings, the unadjusted incidence rate (per 100 person years) for major critical site bleeding was 13.01 and 4.66 for major bleeding requiring hospitalization, respectively [28]. In healthy individuals and individuals with cardiovascular risk factors, ASA increased the risk of major bleeding by 48% (*p* < 0.001). In individuals with diabetes, aspirin increased the risk of bleeding by 49% (*p* = 0.13) [29].

The prevalence of bleeding increases with age, since capillaries become frailer. Ecchymoses in the elderly (senile purpura) is estimated to be present in about 12% of individuals after the age of 50 years and up to 30% after age 75, especially those with fair skin [30]. Common concomitant conditions in this age group further lead to an increased risk both of serious bleedings and of cardiovascular events [31]. In conclusion, there is a significant background incidence of spontaneous bleeding in the elderly. 

Postmarketing data generated from high numbers of heterogenous groups may detect rare adverse effects. However, it is especially challenging to assess the causality of spontaneous reports in general and on bleeding due to pharmacodynamic interactions in particular. First, such reports may not be fully documented. On the grounds of limited information, it is also difficult to assess the causal relationship since confounders must be considered. Lack of control group and reporting bias limit their external validity. Since there is no control group, the effect of EGb 761^®^ cannot be discriminated from the background incidence and from adverse effects of the concomitant medication. Overall, the level of evidence is low, unless confirmed by controlled clinical pharmacodynamic or pharmacokinetic studies. Clinical trials are conducted in highly standardized, controlled situations, and deliver results with high-level internal validity. The laboratory values from the randomized and placebo-controlled study presented here are therefore more appropriate than case reports and database analyses of spontaneous reports when it comes to determining the risk of haemorrhage associated with EGb 761^®^. 

We found that over the 6-month duration of the trial, there was neither an increase in haemorrhage in the treatment groups, nor any alteration in coagulation parameters in either the high- or low-dose EGb 761^®^ treatment group. We found no indication that EGb 761^®^ enhances the antiplatelet effect of ASA nor the anticoagulant activity of warfarin. However, the latter group was small. Our findings in this large-scale double-blind study are in line with other studies, thus confirming that neither the use of EGb 761^®^ alone nor its use with ASA is associated with an increased risk of bleeding. ASA represents the class of platelet aggregation inhibitors, and warfarin belongs to the group of vitamin K antagonists; both drugs act as indirect anticoagulants. So far, only very few data are available on potential interactions between EGb 761^®^ and the class of direct anticoagulants. Postmarketing data do not reveal evidence for such an interaction; however, further research is needed.

## Figures and Tables

**Table 1:** Demographic data shown by concomitant use of anticoagulants and per treatment group.

			Dropouts		Female		Age [Years]		Weight [kg]		Height [cm]		Body Mass Index [kg/m^2^]	
Group	Treatment Groups	ITT [*n*]	[*n*]	[%]	[*n*]	[%]	Mean	SD	Mean	SD	Mean	SD	Mean	SD
Neither ASA nor warfarin	Placebo	99	24	24.2	57	57.6	77.0	7.9	67.6	13.5	164.1	9.8	25.0	4.2
	EGb 761^®^ 120 mg	107	23	21.5	54	50.5	78.1	6.8	68.6	13.8	165.1	10.7	25.1	4.1
	EGb 761^®^ 240 mg	119	20	16.8	75	63.0	78.1	6.8	65.6	13.9	162.2	9.7	24.8	4.1
ASA	Placebo	68 *	13	19.1	30	44.1	78.2	6.7	72.0	13.0	166.3	10.6	26.0	3.9
	EGb 761^®^ 120 mg	57	10	17.5	29	50.9	79.4	7.0	69.4	16.1	162.5	10.8	26.2	5.1
	EGb 761^®^ 240 mg	48 **	10 #	20.8	21	43.8	78.0	7.6	72.6	13.9	166.1	9.4	26.2	3.8
Warfarin	Placebo	9 *	2	22.2	3	33.3	78.6	7.2	73.2	10.4	171.2	7.9	24.9	2.6
	EGb 761^®^ 120 mg	5	1	20.0	1	20.0	81.0	9.3	72.6	18.6	173.2	11.5	24.1	5.0
	EGb 761^®^ 240 mg	4 **	1 #	25.0	1	25.0	80.0	8.3	82.5	14.1	172.0	11.5	27.8	3.2

* 2 patients took ASA and warfarin concomitantly, ** 1 patient took ASA and warfarin concomitantly. ITT = Intention to treat analysis set, *n* sample size, ASA acetylsalicylic acid, # 1 drop out took ASA and Warfarin concomitantly.

**Table 2:** Coagulation parameters and bleeding time of patients taking neither acetylsalicylic acid nor warfarin.

					95% CI				Difference Placebo–EGb			Difference EGb 120 mg–EGb 240 mg		
Visit	Treatment	*N*	Mean	SD	LL	UL	Min	Max	Mean	SD	*p*	Mean	SD	*p*
PT [s]														
Baseline	Placebo	93	10.50	0.81	10.33	10.67	9.20	13.50						
	EGb 761^®^ 120 mg	105	10.51	0.73	10.37	10.65	9.20	12.80	−0.01	0.77	0.917			
	EGb 761^®^ 240 mg	116	10.44	0.63	10.33	10.56	8.20	11.80	0.06	0.72	0.580	0.07	0.68	0.466
Week 6	Placebo	85	10.59	0.75	10.43	10.75	8.70	13.80						
	EGb 761^®^ 120 mg	99	10.54	0.63	10.41	10.66	9.20	12.40	0.05	0.69	0.617			
	EGb 761^®^ 240 mg	109	10.39	0.63	10.27	10.51	8.60	12.10	0.20	0.69	0.041	0.15	0.63	0.083
Week 26	Placebo	82	10.46	0.87	10.27	10.65	8.80	13.80						
	EGb 761^®^ 120 mg	95	10.41	0.93	10.22	10.60	8.80	16.70	0.05	0.91	0.701			
	EGb 761^®^ 240 mg	106	10.33	0.77	10.19	10.48	7.00	12.40	0.13	0.81	0.289	0.07	0.85	0.536
INR														
Baseline	Placebo	93	1.02	0.08	1.00	1.04	0.90	1.30						
	EGb 761^®^ 120 mg	105	1.02	0.07	1.00	1.03	0.90	1.20	0.00	0.08	0.768			
	EGb 761^®^ 240 mg	116	1.01	0.06	1.00	1.02	0.80	1.10	0.01	0.07	0.410	0.00	0.07	0.580
Week 6	Placebo	85	1.03	0.07	1.01	1.04	0.80	1.30						
	EGb 761^®^ 120 mg	99	1.02	0.07	1.01	1.04	0.90	1.20	0.00	0.07	0.721			
	EGb 761^®^ 240 mg	109	1.01	0.07	0.99	1.02	0.80	1.20	0.02	0.07	0.057	0.02	0.07	0.093
Week 26	Placebo	82	1.02	0.09	1.00	1.03	0.90	1.30						
	EGb 761^®^ 120 mg	95	1.01	0.09	1.00	1.03	0.90	1.60	0.00	0.09	0.934			
	EGb 761^®^ 240 mg	106	1.00	0.08	0.99	1.02	0.70	1.20	0.01	0.08	0.216	0.01	0.08	0.251
APTT [s]														
Baseline	Placebo	95	27.03	2.85	26.45	27.61	22.00	36.00						
	EGb 761^®^ 120 mg	105	27.45	2.69	26.93	27.97	23.00	39.00	−0.42	2.77	0.290			
	EGb 761^®^ 240 mg	116	27.58	3.18	26.99	28.16	19.00	41.00	−0.55	3.04	0.195	−0.13	2.96	0.745
Week 6	Placebo	85	27.72	2.81	27.11	28.32	20.00	36.00						
	EGb 761^®^ 120 mg	99	27.37	2.23	26.93	27.82	23.00	33.00	0.34	2.52	0.357			
	EGb 761^®^ 240 mg	110	27.91	2.97	27.35	28.47	22.00	35.00	−0.19	2.90	0.649	−0.54	2.65	0.146
Week 26	Placebo	82	27.43	2.65	26.84	28.01	21.00	33.00						
	EGb 761^®^ 120 mg	95	27.72	2.72	27.16	28.27	22.00	40.00	−0.29	2.69	0.477			
	EGb 761^®^ 240 mg	106	27.51	2.95	26.94	28.08	21.00	35.00	−0.08	2.83	0.843	0.21	2.85	0.609
Bleeding time [min]														
Baseline	Placebo	56	4.28	2.03	3.74	4.82	0.40	10.00						
	EGb 761^®^ 120 mg	61	4.40	1.62	3.99	4.82	0.50	8.00	−0.12	1.82	0.717			
	EGb 761^®^ 240 mg	58	4.82	1.91	4.32	5.33	1.30	9.50	−0.54	1.97	0.145	−0.42	1.77	0.199
Week 6	Placebo	50	3.95	1.91	3.41	4.49	0.30	10.50						
	EGb 761^®^ 120 mg	59	4.41	1.49	4.03	4.80	2.00	8.00	−0.47	1.69	0.156			
	EGb 761^®^ 240 mg	56	4.45	1.55	4.03	4.87	2.00	9.50	−0.50	1.73	0.139	−0.04	1.52	0.898
Week 26	Placebo	47	4.30	1.55	3.84	4.76	0.90	7.30						
	EGb 761^®^ 120 mg	59	3.98	1.76	3.52	4.44	0.30	11.00	0.32	1.67	0.327			
	EGb 761^®^ 240 mg	44	4.45	1.97	3.85	5.04	1.00	9.30	−0.15	1.77	0.696	−0.47	1.85	0.208

*N* sample size, SD standard deviation, 95% CI 95% confidence interval, LL lower limit, UL upper limit, MIN minimum, MAX maximum, *p* *p*-value of two sided *t*-test, PT prothrombin time, INR international normalized ratio, APTT activated partial thromboplastin time.

**Table 3:** Coagulation parameters and bleeding time of patients taking acetylsalicylic acid.

					95% CI				Difference Placebo–EGb 761			Difference EGb 120 mg–EGb 240 mg		
Visit	Treatment	*N*	Mean	SD	LL	UL	MIN	MAX	Mean	SD	*p*	Mean	SD	*p*
PT [s]														
Baseline	Placebo	67	11.42	5.30	10.13	12.72	9.40	44.20						
	EGb 761^®^ 120 mg	53	10.51	0.74	10.31	10.71	8.40	12.00	0.91	3.99	0.215			
	EGb 761^®^ 240 mg	48	10.63	0.66	10.44	10.82	9.30	13.00	0.79	4.07	0.304	−0.12	0.70	0.392
Week 6	Placebo	62	10.83	2.14	10.29	11.38	9.10	26.20						
	EGb 761^®^ 120 mg	50	10.52	0.71	10.32	10.72	9.10	12.50	0.31	1.66	0.323			
	EGb 761^®^ 240 mg	46	10.79	0.61	10.61	10.97	9.40	12.30	0.04	1.67	0.896	−0.27	0.67	0.049
Week 26	Placebo	57	10.93	2.70	10.22	11.65	8.90	26.20						
	EGb 761^®^ 120 mg	49	10.41	0.75	10.19	10.62	8.10	11.90	0.53	2.05	0.189			
	EGb 761^®^ 240 mg	46	10.58	0.76	10.36	10.81	9.10	13.40	0.35	2.07	0.395	−0.18	0.75	0.256
INR														
Baseline	Placebo	67	1.12	0.52	0.99	1.24	0.90	4.30						
	EGb 761^®^ 120 mg	53	1.02	0.08	1.00	1.04	0.80	1.20	0.10	0.39	0.162			
	EGb 761^®^ 240 mg	48	1.02	0.07	1.00	1.04	0.90	1.30	0.10	0.40	0.200	−0.00	0.07	0.789
Week 6	Placebo	61	1.05	0.20	1.00	1.10	0.90	2.50						
	EGb 761^®^ 120 mg	50	1.02	0.07	1.00	1.04	0.90	1.20	0.03	0.16	0.254			
	EGb 761^®^ 240 mg	46	1.05	0.06	1.03	1.06	0.90	1.20	0.01	0.16	0.868	−0.03	0.07	0.037
Week 26	Placebo	57	1.06	0.25	0.99	1.13	0.90	2.50						
	EGb 761^®^ 120 mg	49	1.01	0.07	0.99	1.03	0.80	1.10	0.05	0.19	0.207			
	EGb 761^®^ 240 mg	46	1.03	0.07	1.00	1.05	0.90	1.30	0.03	0.20	0.414	−0.02	0.07	0.291
APTT [s]														
Baseline	Placebo	67	28.78	5.54	27.43	30.13	22.00	61.00						
	EGb 761^®^ 120 mg	53	27.68	2.96	26.86	28.50	23.00	37.00	1.10	4.58	0.196			
	EGb 761^®^ 240 mg	48	27.29	3.00	26.42	28.16	22.00	41.00	1.48	4.65	0.094	0.39	2.98	0.515
Week 6	Placebo	62	28.18	3.01	27.41	28.94	23.00	42.00						
	EGb 761^®^ 120 mg	50	27.38	3.33	26.43	28.33	24.00	42.00	0.80	3.15	0.186			
	EGb 761^®^ 240 mg	46	27.85	3.15	26.91	28.78	23.00	41.00	0.33	3.07	0.582	−0.47	3.24	0.482
Week 26	Placebo	57	28.61	3.36	27.72	29.51	24.00	42.00						
	EGb 761^®^ 120 mg	49	27.55	2.82	26.74	28.36	23.00	36.00	1.06	3.12	0.083			
	EGb 761^®^ 240 mg	46	27.59	3.37	26.59	28.59	21.00	42.00	1.03	3.36	0.127	−0.04	3.10	0.955
Bleeding time [min]														
Baseline	Placebo	37	5.13	2.23	4.39	5.87	1.50	13.00						
	EGb 761^®^ 120 mg	32	4.71	2.78	3.71	5.71	1.40	15.00	0.42	2.50	0.489			
	EGb 761^®^ 240 mg	29	5.12	1.71	4.47	5.78	2.10	9.00	0.01	2.02	0.991	−0.41	2.34	0.491
Week 6	Placebo	33	4.61	1.88	3.95	5.28	1.50	9.00						
	EGb 761^®^ 120 mg	31	4.05	1.89	3.35	4.74	1.00	8.00	0.57	1.89	0.234			
	EGb 761^®^ 240 mg	28	5.55	2.15	4.72	6.38	2.50	10.00	−0.94	2.01	0.074	−1.50	2.02	0.006
Week 26	Placebo	31	4.57	2.70	3.58	5.56	0.90	16.00						
	EGb 761^®^ 120 mg	28	4.73	2.02	3.95	5.52	2.20	9.50	−0.16	2.40	0.794			
	EGb 761^®^ 240 mg	26	5.14	2.81	4.00	6.27	1.50	12.00	−0.57	2.75	0.438	−0.41	2.43	0.542

*N* sample size, SD standard deviation, 95% CI 95% confidence interval, LL lower limit, UL upper limit, MIN minimum, MAX maximum, *p p*-value of two sided *t*-test, PT prothrombin time, INR international normalized ratio, APTT activated partial thromboplastin time.

**Table 4:** Coagulation parameters of patients taking warfarin.

		Placebo			EGb 761 120 mg			EGb 761 240 mg			Comparison EGb (Both Doses) vs. Placebo
	Visit	*N*	Mean [Median]	(SD)	*N*	Mean [Median]	(SD)	*N*	Mean [Median]	(SD)	*p*-Value
PT [s]	Baseline	9	26.8 [19.8]	(15.8)	5	24.1 [21.9]	(11.1)	4	16.9 [16.7]	(4.9)	0.346
	Week 26	7	24.1 [26.1]	(5.1)	4	23.2 [23.1]	(2.4)	4	17.4 [16.8]	(3.8)	0.136
APTT [s]	Baseline	9	40.4 [36.0]	(11.3)	5	34.6 [32.0]	(6.1)	4	35.5 [33.5]	(6.1)	0.216
	Week 26	7	38.7 [40.0]	(7.0)	4	34.5 [35.0]	(3.0)	4	36.5 [37.0]	(5.5)	0.174
INR	Baseline	9	2.56 [1.90]	(1.47)	5	2.34 [2.10]	(1.09)	4	1.63 [1.65]	(0.46)	0.367
	Week 26	7	2.26 [2.40]	(0.46)	4	2.20 [2.25]	(0.22)	4	1.65 [1.609]	(0.34)	0.157

*N* sample size, PT prothrombin time, INR international normalized ratio, APTT activated partial thromboplastin time.

**Table 5:** Bleeding events documented as adverse events by treatment.

	Placebo	EGb 761 120 mg	EGb 761 240 mg	Total
Ecchymosis	9	4	7	20
Epistaxis	5	0	1	6
Faecal occult blood positive	0	0	1	1
Gastric ulcer haemorrhage	0	0	1	1
Haematuria present	0	1	2	3
Rectal bleeding	0	4	1	5
Retinal haemorrhage	0	1	0	1
Total	14	10	13	37

## Data Availability

Raw data from this study cannot be provided, because this was not covered by the patients’ informed consent when the study was conducted.
